# Overexpression of multiple* cytochrome P450* genes with and without knockdown resistance mutations confers high resistance to deltamethrin in *Culex quinquefasciatus*

**DOI:** 10.1186/s40249-024-01269-2

**Published:** 2025-01-13

**Authors:** Saowanee Chamnanya, Benyapa Kiddela, Jassada Saingamsook, Woottichai Nachaiwieng, Nongkran Lumjuan, Pradya Somboon, Jintana Yanola

**Affiliations:** 1 Center of Veterinary Medical Diagnostic and Animal Health Innovation, Faculty of Veterinary Medicine, Chiang Mai University, Chiang Mai, 50100 Thailand; 2 Department of Medical Technology, Faculty of Associated Medical Sciences, Chiang Mai University, Chiang Mai, 50200 Thailand; 3 Department of Parasitology, Faculty of Medicine, Chiang Mai University, Chiang Mai, 50200 Thailand; 4 School of Health Science, Mae Fah Luang University, Chiang Rai, 57100 Thailand; 5 Biomedical Technology Research Group for Vulnerable Populations, Mae Fah Luang University, Chiang Rai, 57100 Thailand; 6 Research Institute for Health Sciences, Chiang Mai University, Chiang Mai, 50200 Thailand

**Keywords:** *Culex quinquefasciatus*, Insecticide resistance, Pyrethroid, Deltamethrin, Cytochrome P450s, Knockdown resistance (*kdr)*, *Voltage-gated sodium channel (vgsc)*

## Abstract

**Background:**

The cytochrome P450s-mediated metabolic resistance and the target site insensitivity caused by the knockdown resistance (*kdr*) mutation in the *voltage-gated sodium channel* (*vgsc*) gene were the main mechanisms conferring resistance to deltamethrin in *Culex quinquefasciatus* from Thailand. This study aimed to investigate the expression levels of *cytochrome P450* genes and detect mutations of the *vgsc* gene in deltamethrin-resistant *Cx. quinquefasciatus* populations in Thailand.

**Methods:**

Two field-collected strains of *Cx. quinquefasciatus*, Cq_SP and Cq_NiH, were selected with deltamethrin to generate the resistant strains Cq_SP-R and Cq_NiH-R, respectively. Bioassays were tested on larvae and adults of each strain according to WHO methods. Eight *cytochrome P450* genes were analyzed for the expression level using quantitative real time-PCR. The cDNA of mosquitoes was amplified and sequenced for four fragments of *vgsc* gene. The *kdr* L1014F mutation and the haplotype of the *CYP9M10* gene were detected in survivor and dead mosquitoes after exposure to the deltamethrin WHO test paper. Statistical analyses were performed using Fisher’s exaction test.

**Results:**

Bioassay tests revealed a significantly higher resistance level in Cq_SP-R than in Cq_NiH-R strains in both larvae and adults. All eight *cytochrome P450* genes were significantly overexpressed in larvae of Cq_NiH-R strain compared to the parent and susceptible Cq_Sus strains. The *CYP6AA7* and *CYP9J34* genes had the highest expression ratios, exceeding 24-fold in Cq_NiH-R larvae. In Cq_SP-R strain, the *CYP4H34* and *CYP9J34* genes were overexpressed in both stages*.* The *kdr* L1014F mutation was found in Cq_SP-R and its parent Cq_SP strains with a significantly higher mutant allele frequency in the survivor mosquitoes than in dead mosquitoes (*P* < 0.0001). The V240M and novel L925F mutations were found only in Cq_SP-R strain. Heterozygous genotype for the D-Cu( +)/Cu(–) of *CYP9M10* gene was detected in Cq_NiH and Cq_NiH-R strains but other strains were mostly homozygous for the Cu(–)/Cu(–).

**Conclusions:**

Overexpression of multiple *cytochrome P450* genes alone has a relatively minor impact on resistance. The combined mechanisms of *cytochrome P450*- and *kdr*-mediated resistance result in significantly higher resistance to deltamethrin in *Cx. quinquefasciatus*. This study supports sustainable public health initiatives in Thailand to address the evolving challenges of insecticide resistance.

## Background

The southern house mosquito, *Culex quinquefasciatus*, is the primary vector for several pathogens especially including the West Nile virus and the parasitic *Wuchereria bancrofti* nematode (agent of bancroftian filariasis) [1]. Bancroftian filariasis is endemic along the Thailand-Myanmar border, with a high incidence among Myanmar migrant workers in Thailand [2, 3]. The influx of Myanmar migrant workers in Thailand has raised concerns due to the potential transmission of bancroftian filariasis, aided by the widespread breeding of *Cx. quinquefasciatus* in urban areas [4]. In Thailand, the government implements a lymphatic filariasis (LF) surveillance program on a biennial basis, primarily targeting immigrants [2]. Mosquito control is the primary strategy for mitigating the transmission of mosquito-borne diseases, often involving the use of insecticides to manage vector density in endemic areas or during epidemic outbreaks [5, 6]. Pyrethroid insecticides have become increasingly important in mosquito control initiatives globally, including Thailand [7, 8]. However, their extensive use has led to the emergence of insecticide resistance in *Aedes* and *Culex* mosquitoes, representing a pressing concern [9]. High level of resistance to pyrethroid insecticides in *Cx. quinquefasciatus* has been documented across Thailand [4, 10].

Major insecticide resistance mechanisms, including metabolic, target site and cuticular resistance, play a role in mosquito resistance to pyrethroid insecticides [11]. Metabolic resistance is due to the increased activity of metabolic enzymes, such as cytochrome P450 monooxygenases, which can metabolize pyrethroid, leading to lower toxicity and more efficient excretion of the active ingredients, particles pyrethroid [11]. Many insects rely heavily on the overexpression of *cytochrome P450* (*CYP450*) genes to detoxify xenobiotics from their bodies [12]. Cytochrome P450s are a large superfamily where the CYP4, CYP6 and CYP9 families play an important role in the biodegradation of insecticide particles in insecticide resistance in mosquitoes, including *Cx. quinquefasciatus* [13–15]. Target-site resistance, known as knockdown resistance (*kdr*), is attributed to mutations in the *voltage-gated sodium channel* (*vgsc*) gene within the protein-coding regions [16]. As this reduces the ability of the pyrethroid to affect its target VGSC protein, target-site resistance often results in high levels of pyrethroid resistance. The common *kdr* L1014F mutation conferred resistance to pyrethroid in the *Cx. quinquefasciatus* populations from several countries including Thailand [10, 17–19].

In Thailand, there have been frequent reports of *Cx. quinquefasciatus* developing resistance to pyrethroid [4, 10, 19]. Two primary mechanisms, specifically the *kdr* L1014F mutation and cytochrome P450 monooxygenases, have been identified as being responsible for deltamethrin resistance in the Thai *Cx. quinquefasciatus* population [4]. The *kdr* L1014F mutation, in particular, is widespread at a high frequency throughout Thailand [19]. This study aimed to assess the resistance levels of *Cx. quinquefasciatus* to deltamethrin conferred by *CYP450*-mediated resistance, both independently and in combination with the *kdr* L1014F mutations. We focus on analyzing the expression levels of *CYP450* genes and identifying mutations in the *vgsc* gene that contribute to deltamethrin resistance in *Cx. quinquefasciatus* populations in Thailand. The findings of this study have direct implications for implementing resistance management strategies against populations carrying these resistance mechanisms, either individually or in combination.

## Methods

### Mosquito strain and deltamethrin selection

Five strains of *Cx. quinquefasciatus,* including two field-collected parental strains (Cq_SP and Cq_NiH), two deltamethrin-selected strains (Cq_SP-R and Cq_NiH-R), and a reference susceptible Cq_Sus strain, were used in this study. All five mosquito strains have been maintained and reared at 25 ± 2 ℃ under a photoperiod of 12:12 (L: D) h in the insectary of the Department of Parasitology, Faculty of Medicine, Chiang Mai University (CMU), Thailand.

Larvae and pupae of *Cx. quinquefasciatus* were collected from the Sri Phum Sub-District, Muang District, Chiang Mai Province, Thailand (18^๐^47′35″ N, 98^๐^58′54′′ E) in 2019 [10] and were subsequently maintained as a colony designated as the Cq_SP strain. The Cq_NiH strain was obtained from the National Institute of Health, Department of Medical Sciences, Ministry of Public Health, Thailand and has been maintained continuously in the CMU insectary since 2015. This strain was a long-established colony originally collected from Pom Prab Satru Phai District, Bangkok, Thailand in 1978 [20]. The susceptible Cq_Sus strain continuously had over 98% mortality in the adult bioassay tested with 0.05% deltamethrin-impregnated WHO paper for over ten generations.

Establishing a deltamethrin-resistant Cq_SP-R strain, the parental Cq_SP strain was subjected to selection pressure with deltamethrin throughout four generations in a controlled laboratory environment. The larvae were exposed to deltamethrin concentrations of 0.18 µg/L, 0.50 µg/L, 1.17 µg/L, and 1.20 µg/L for the first through fourth generations, respectively. These concentrations were determined to effectively induce mortality in 50% of the treated individuals within 24 h. Similarly, the parental Cq_NiH strain was subjected to deltamethrin selection for five generations, leading to the establishment of the deltamethrin-resistant Cq_NiH-R strain. Adults were exposed to 0.05% deltamethrin-impregnated WHO paper in the first two generations. Larvae from the third to fifth generations were then exposed to deltamethrin concentrations of 0.18 µg/L, 0.50 µg/L, and 1.17 µg/L, respectively. Survivors from each generation were retained to continue the selection process.

### Insecticide susceptibility test

The larval susceptibility test was performed following the WHO standard guidelines [21] with slight modifications as previously described [4]. Briefly, the bioassays were carried out using batches of 25 early 4th instar larvae per beaker containing 250 ml of distilled water. Seven to eight different insecticide concentrations (0.05–5 µg/L) giving 0–100% mortality were tested against the larvae. The bioassays were conducted with four replicates for each concentration using a single beaker for each replication. The stock and serial dilutions of deltamethrin (Supelco, Bellefonte, PA, USA) were prepared in ethanol. In the control experiments, 0.4% ethanol was included in 250 ml of water. Larval mortality was recorded after 24 h exposure. Adult susceptibility tests were conducted following WHO standard methods [22, 23]. Due to the high levels of pyrethroid resistance observed in *Cx. quinquefasciatus* in Thailand [4, 10, 19], *Anopheles* mosquito discriminating concentrations were used instead of *Culex* mosquito discriminating concentrations. Four batches of 25, one–two days old, non-blood-fed females were exposed to 0.05% deltamethrin-impregnated paper and 0.75% permethrin-impregnated paper for 60 min in the standard WHO bioassay test tubes. The mortality was scored and recorded after 24 h. Dead (susceptible) and survivor (resistant) mosquitoes after the bioassays were stored at − 20 ℃ until tested.

### Gene expression analysis of the* Culex quinquefasciatus*-*cytochrome P450* genes using quantitative real-time PCR (qRT-PCR)

The expression levels of the candidate *cytochrome P450* genes, *CYP4C52v1*, *CYP4H34*, *CYP6AA7*, *CYP6P14*, *CYP9AL1*, *CYP9J34*, *CYP9J45*, and *CYP9M10*, were measured using quantitative real-time PCR (qRT-PCR). Those candidate genes were selected for expression analysis based on previous evidence indicating their involvement in metabolic resistance [13–15]. The total RNA was isolated from the fourth instar larvae and one-day-old females of each mosquito strain. The pool of ten mosquitoes was used to extract total RNA for three replications using the illustra^™^ RNAspin Mini Isolation Kit (GE Healthcare, Buckinghamshire, UK), following the manufacturer’s instruction. The total RNA was reversed transcribed to single-strand cDNA using the SuperScript^™^ III First-Strand Synthesis system (Invitrogen, Carlsbad, CA, USA). The qRT-PCR was performed with the SensiFAST^™^ SYBR^®^ Lo-ROX Kit (Bioline, Meridian Bioscience, Germany) in the 7500 Fast Real-Time PCR machine (Applied Biosystems, USA). Each qRT-PCR reaction was carried out in 25 µl final volume containing 1 × SYBR^®^ Green master mix, 1 µl of cDNA, and the *cytochrome P450* genes specific primer pairs [24] at a final concentration of 5 µmol/L. All samples and negative control were performed in triplicate. The qRT-PCR reaction cycle consists of a denaturing step of 50 ℃ for 2 min, then 95 ℃ for 10 min, followed by 40 cycles of 95 ℃ for 15 s and 60 ℃ for 1 min. The specificity of the PCR reactions was assessed by a melting curve analysis using Dissociation Curves software. Relative expression levels for the *cytochrome P450* genes were calculated by the 2^−∆∆CT^ method using 7500 software v2.3 (Applied Biosystems, USA). The expression of the target gene was normalized using the *18S ribosome RNA* gene as an endogenous control [24].

### Amplification and DNA sequencing of the fragments of the *Culex quinquefasciatus-vgsc* gene

**Fig. 1:**
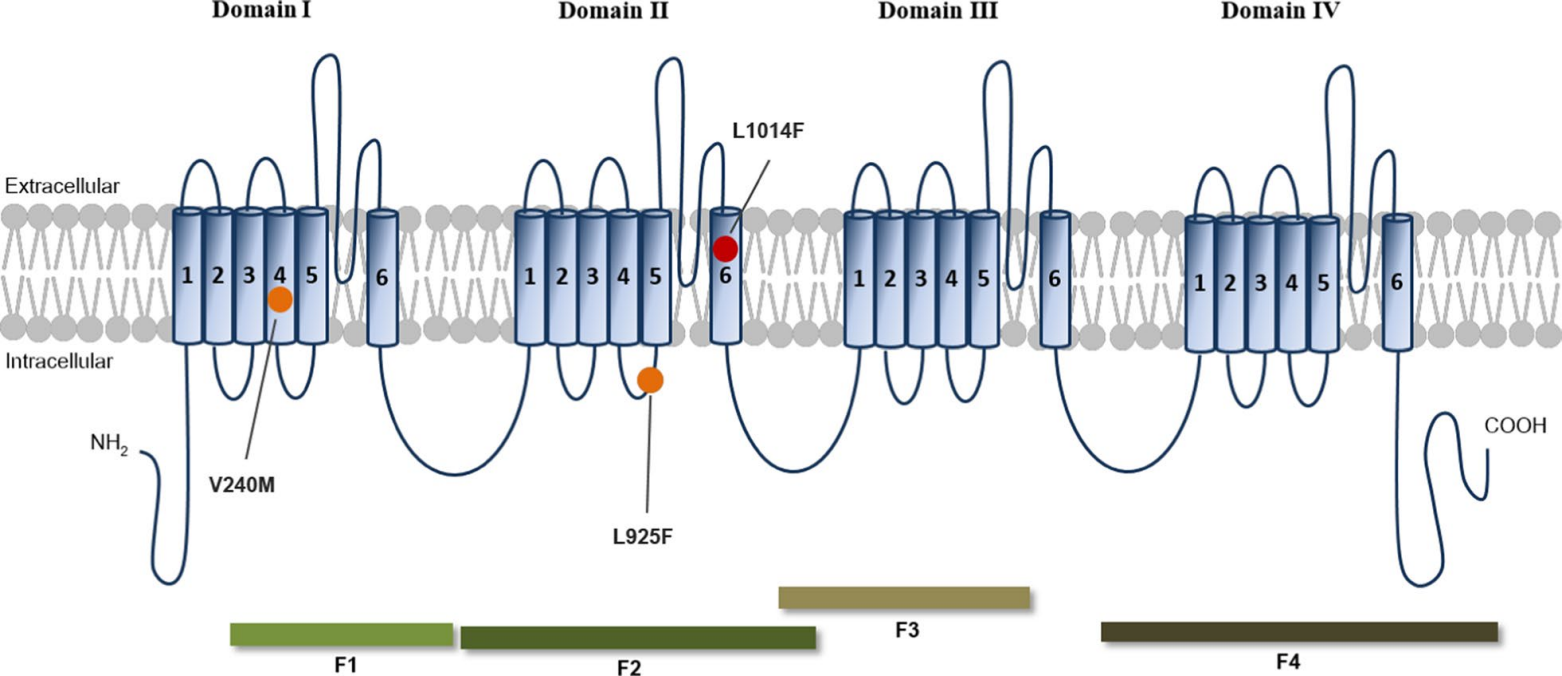
Schematic of the voltage-gated sodium channel. The VGSC pore-forming α-subunit consists of four homologous repeat domains (I–IV), each with six transmembrane segments (S1–S6) connected by intracellular and extracellular loops. The four fragments of the *vgsc* gene, including the IS2–IS6 region of domain I (green fragment; F1), the IIS1–IIS6 region of domain II (dark green fragment; F2), the IIIS1–IIIS6 region of domain III (tan fragment; F3) and the IVS1–IVS6 region of domain IV (dark tan fragment; F4) were amplified in this study. Three non-synonymous mutations in the *vgsc* gene were identified in the resistant strains of *Cx. quinquefasciatus*. These *vgsc* mutations, including V240M, novel L925F and *kdr* L1014F, were indicated by the red and orange circles. The red circle denotes the *kdr* L1014F mutation observed in both the Cq_SP and Cq_SP-R strains. The orange circle represents two *vgsc* mutations, V240M and L925F, exclusively found in the Cq_SP-R strain

The total RNA was isolated from a single and pooled ten mosquitoes of the one-day-old females of each strain for three replications. The single-strand cDNA was synthesized and used as a template for PCR amplification. Four fragments of the *Cx. quinquefasciatus-vgsc* gene were amplified for encompassing four domains of VGSC protein (Fig. 1) using four pairs of primer, newly designed in this study (Table 1). All PCR reactions were carried out in a volume of 20 µl that contained a final concentration of 1.25 units of Platinum *Taq* DNA Polymerase High Fidelity (Invitrogen, Carlsbad, CA, USA), 1 × High Fidelity PCR buffer, 0.5 mol/L dNTPs, 2.5 mol/L MgSO_4_ and 0.5 µmol/L each of the forward and reverse primers. The amplifications consisted of an initial heat activation step at 94 ℃ for 90 s, followed by 35 cycles of 94 ℃ for 45 s, 62 ℃ for 45 s, and 68 ℃ for 60 s with a final extension step at 68 ℃ for 10 min. PCR products were analyzed by electrophoresis on 2% agarose gel (Invitrogen, Carlsbad, CA, USA) using a voltage of 120 v for 25 min and visualized under UV light by RedSafe^™^ Nucleic Acid Staining (iNtRON biotechnology, Korea). The PCR products were purified using illustra™ ExoStar™ 1-Step kit (GE Healthcare, Buckinghamshire, UK). The PCR product was purified and then sent to Macrogen (Seoul, Korea) for DNA sequencing in both directions.

**Table 1:** Sequences of primers for amplifying the *Culex quinquefasciatus voltage-gated sodium channel* gene

Primer name	Primer sequence (5’- 3’) ^a^	PCR product size (bp)	Domain region in VGSC protein
Cq_IS2-IS6_F Cq_IS2-IS6_R	AGTGATGGCGCGAGGTTTCA TTTCGCTGCGGCCGCTTCTT	790	IS2–IS6
Cq_IIS1-IIS6_F Cq_IIS1-IIS6_R	ACGAGGACGGTCCGACGTTCAA TGCACGGACGCAATCTGGCT	970	IIS1–IIS6
Cq_IIIS1-IIIS6_F Cq_IIIS1-IIIS6_R	AGAAAAGCGCGACGCCAGCA TGTTGGTTTCGCGGATGGGC	956	IIIS1–IIIS6
Cq_IVS1-IVS6_F Cq_IVS1-IVS6_R	AAGAAGGCTGGGGGATCGCT TCCGGATCGAACTGCTGCCA	974	IVS1–IVS6

^a^Primers for amplifying the *Cx. quinquefasciatus*-*vgsc* gene fragments were designed using sequences from the Johannesburg strain of *Cx. quinquefasciatus,* VectorBase Transcript ID CPIJ007595 [25]. Four pairs of specific primers were designed to encompass the VGSC protein region (domain I–IV) with putative non-synonymous mutations of the *Cx. quinquefasciatus-vgsc* gene [26] and used to amplify the DNA fragments.

### Genotyping of the *CYP9M10 *gene and the *kdr* L1014F mutation in *vgsc* gene

After exposure to 0.05% deltamethrin WHO paper, the survivor and dead mosquitoes were randomly selected for genotyping. Genomic DNA was extracted from a single mosquito using the method previously described [27]. Genotyping of the *CYP9M10* gene was performed using PCR I and II, previously described [28], with some modifications. In brief, the PCR reaction was carried out in a 25 μl PCR master mix that contained 1.5 mol/L MgCl_2_, 1 × PCR buffer, 0.2 µmol/L of each primer, 200 µmol/L dNTP, 0.2 U/µl Platinum *Taq* DNA polymerase (Invitrogen, Carlsbad, CA, USA). Each PCR reaction was performed in a 25 μl reaction volume. The PCR master mix contains 1 × PCR buffer, 1.5 mol/L MgCl_2_, 200 µmol/L dNTP, 0.2 U/μl of Platinum *Taq* DNA polymerase (Invitrogen, Carlsbad, CA, USA), 0.2 µmol/L each primer of *Gen*1F and *Gen*1R for genotyping PCR I, and Gen2Fa and Gen2Fb for genotyping PCR II, and 0.4 µmol/L of Gen2R for genotyping PCR II. The PCR reaction began with a 2-min denaturation step at 94 ℃, followed by 35 cycles of 30 s at 50 ℃, 30 s at 72 ℃, and 30 s at 72 ℃, with a 2-min extension step at 72 ℃. PCR products were analyzed by electrophoresis on 2% agarose gel (Invitrogen, Carlsbad, CA, USA) using a voltage of 100 v for 30 min and visualized under UV light by RedSafe^™^ Nucleic Acid Staining (iNtRON biotechnology, Korea). The *kdr* L1014F mutations in the *Cx. quinquefasciatus-vgsc* gene was detected using the tetra-primer AS-PCR and real-time PCR with melt curve analysis methods as described previously [19, 29].

### Data analysis

The concentration-mortality responses for the larvae susceptibility test were determined by probit analysis [30] using the LdP Line software (www.Ehabsoft.com/LDPline). The statistical significance of the gene expressions was calculated using a Student’s *t*-test for all 2-sample comparisons and one-way analysis of variance (ANOVA) for multiple sample comparisons using SPSS version 17.0 (SPSS Inc., Chicago, USA); a value of *P* ≤ 0.05 was considered statistically significant. Significant overexpression was determined using a cut-off value of a ≥ twofold change in expression [31]. Nucleotide sequences of the *vgsc* gene were aligned using Geneious Prime software version 2023.1.2 (Biomatters, Auckland, New Zealand). The genotypes and allele frequencies of the *CYP9M10* gene and the *kdr* L1014F mutation in the *vgsc* gene of *Cx. quinquefasciatus* mosquitoes were calculated, and statistical differences between the survivor and dead mosquito groups were examined by Fisher’s exaction test using Genepop [32].

## Results

### Toxicity of pyrethroid to the parental and selected resistant strains of *Culex quinquefasciatus*

The larvae bioassay test of deltamethrin in the parental Cq_SP and Cq_NiH strains showed the levels of lethal concentration 50 (LC_50_) of 0.440 µg/L and 0.080 µg/L, respectively. The adult bioassay test of the parental Cq_SP and Cq_NiH strains indicated mortalities of 26.5 and 55.0%, respectively, after exposure to 0.05% deltamethrin-impregnated papers (Table 2). Following four generations of selection on the parental Cq_SP strain using deltamethrin in larval bioassays, the Cq_SP-R strain exhibited an increased LC_50_ level of 2.530 µg/L, corresponding to a resistance ratio of 316.2 compared to the Cq_Sus strain. The mortality rate in the adult bioassay of the Cq_SP-R strain was reduced to 0.0% after exposure to 0.05% deltamethrin-impregnated papers (Table 2). After five generations of selection on the parental Cq_NiH strain, the deltamethrin larval bioassay of the Cq_NiH-R strain revealed the LC_50_ of 0.480 µg/L and the resistance ratio of 60. The mortality rate in the adult bioassay of the Cq_NiH-R strain dramatically decreased to 16.1% after exposure to 0.05% deltamethrin-impregnated papers (Table 2). In addition, the female *Cx. quinquefasciatus* of each strain was evaluated using 0.75% permethrin-impregnated paper. The mortality rates of the parental strains, Cq_SP (52.9%) and Cq_NiH (71.0%), exhibited a noticeable decline in the resistant strains Cq_SP-R and Cq_NiH-R, showing 18.2 and 44.3%, respectively (Table 2).

**Table 2:** Toxicity of pyrethroid against the *Culex quinquefasciatus* strains using larval and adult bioassays

Mosquito strains	Larval bioassay^a^				Adult bioassay^c^	
	LC_50_ (µg/L) (tested number)	*χ*^2^ (df)	Slope (± SE)	RR^b^	Deltamethrin %Mortality (tested number)	Permethrin %Mortality (tested number)
Cq_SP	0.440 (*n* = 700)	1.000 (4)	2.21 (± 0.09)	55.0	26.5 (*n* = 162)	52.9 (*n* = 153)
Cq_SP-R	2.530 (*n* = 700)	1.000 (3)	1.77 (± 0.11)	316.2	0.0 (*n* = 116)	18.2 (*n* = 122)
Cq_NiH	0.080 (*n* = 700)	1.000 (3)	2.32 (± 0.09)	10.0	55.0 (*n* = 300)	71.0 (*n* = 389)
Cq_NiH-R	0.480 (*n* = 700)	1.000 (2)	3.10 (± 0.08)	60.0	16.1 (*n* = 186)	44.3 (*n* = 210)
Cq_Sus	0.008 (*n* = 700)	1.000 (3)	1.41 (± 0.14)	1.0	98.6 (*n* = 144)	100.0 (*n* = 128)

^a^Larval mosquitoes were tested with deltamethrin.

^b^Resistance ratio compared to susceptible strain (Cq_Sus).

^c^Adult mosquitoes were tested using standard WHO-impregnated paper with 0.05% deltamethrin and 0.75% permethrin.

### Expression levels of *cytochrome P450* genes in *Culex quinquefasciatus*

Eight candidate *cytochrome P450* genes, namely *CYP4C52v1*, *CYP4H34*, *CYP6AA7*, *CYP6P14*, *CYP9AL1*, *CYP9J34*, *CYP9J45,* and *CYP9M10*, were investigated for the gene expression levels in both larval and adult stages of the *Cx. quinquefasciatus* mosquitoes using qRT-PCR analysis. The relative gene expression of each *cytochrome P450* gene exhibited variability across developmental stages (larvae and adult) and among different strains of *Cx. quinquefasciatus* mosquitoes, specifically the Cq_SP and Cq_SP-R strains (Table 3), as well as the Cq_NiH and Cq_NiH-R (Table 4). The relative gene expression ratios with a cut-off value of ≥ twofold higher in the deltamethrin-resistant selected mosquitoes (Cq_SP-R and Cq_NiH-R) compare to their parental strains (Cq_SP and Cq_NiH) and the susceptible strain (Cq_Sus) were considered indicative of significant overexpression of *cytochrome P450* genes (Tables 3–4). All eight *cytochrome P450* genes were found to be overexpressed in the larvae of the Cq_NiH-R strain. In the adult stage, five of eight genes, *CYP6AA7*, *CYP6P14*, *CYP9AL1*, *CYP9J45*, and *CYP9M10*, showed overexpression. Interestingly, the *CYP6AA7* and *CYP6P14* genes were highly overexpressed with an expression ratio of > tenfold in the larval and adult stages of the Cq_NiH-R strain (Table 4). In the Cq_SP-R strain, the *CYP4H34* and *CYP9J34* genes were overexpressed in both stages, but three genes, *CYP9AL1*, *CYP9J45*, and *CYP9M10* revealed an overexpression only in larvae (Table 3)*.*

**Table 3:** The relative level of gene expression of *cytochrome P450* genes in both larvae and adult stages of the deltamethrin selected Cq_SP-R and its parental Cq_SP strains

Stage of mosquito	Transcript ID^a^	Gene	Relative gene expression ± SE^b^		Ratio^c^	*P*-value^*d*^
			Cq_SP (parental strain)	Cq_SP-R (selected strain)		
*Cytochrome P450* genes involved in up-regulation						
Larva	CPIJ011127	CYP4H34* ^#^	0.16 ± 0.08	0.93 ± 0.03	5.9	< 0.0001
	CPIJ005959	CYP6AA7*	0.82 ± 0.08	1.78 ± 0.03	2.2	< 0.0001
	CPIJ005955	CYP6P14*	1.78 ± 0.08	3.84 ± 0.02	2.2	< 0.0001
	CPIJ012470	CYP9AL1*	0.44 ± 0.07	1.61 ± 0.06	3.6	< 0.0001
	CPIJ010546	CYP9J34* ^#^	0.41 ± 0.09	2.99 ± 0.05	7.3	< 0.0001
	CPIJ010537	CYP9J45*	0.20 ± 0.07	0.62 ± 0.05	3.2	< 0.0001
	CPIJ014218	CYP9M10*	0.10 ± 0.11	0.55 ± 0.08	5.5	< 0.0001
Adult	CPIJ011127	*CYP4H34*^#^	0.24 ± 0.05	0.98 ± 0.05	4.0	< 0.0001
	CPIJ010546	*CYP9J34*^#^	0.16 ± 0.06	0.77 ± 0.07	4.9	< 0.0001
*Cytochrome P450* genes involved in down-regulation						
Larva	CPIJ018943	*CYP4C52v1*	19.18 ± 0.06	14.84 ± 0.07	− 1.3	< 0.0001
Adult	CPIJ018943	*CYP4C52v1*	0.72 ± 0.13	0.09 ± 0.04	− 8.1	< 0.0001
	CPIJ005959	*CYP6AA7*	2.79 ± 0.10	0.44 ± 0.05	− 6.4	< 0.0001
	CPIJ005955	*CYP6P14*	2.36 ± 0.11	0.42 ± 0.04	− 5.6	< 0.0001
	CPIJ012470	*CYP9AL1*	0.56 ± 0.09	0.19 ± 0.04	− 2.9	< 0.0001
	CPIJ010537	*CYP9J45*	1.56 ± 0.07	0.06 ± 0.05	− 25.2	< 0.0001
	CPIJ014218	*CYP9M10*	0.36 ± 0.10	0.14 ± 0.04	− 2.5	< 0.0001

^a^The transcript ID number from the vectorbase of the *Cx. quinquefasciatus* genome sequence (http://cquinquefasciatus.vectorbase.org/)

^b^The relative level of gene expression represents the ratio of the gene expression in each resistant strain compared with that in the susceptible Cq_Sus strain. The relative level of gene expression for Cq_Sus is 1.0

^c^The ratio of the relative gene expression in each deltamethrin selected strain compared its parental strain

^d^The independent samples *t*-test is used to calculate the difference in relative gene expression between the deltamethrin selected strain and its parental strain using SPSS version 17.0 (SPSS Inc., Chicago, USA) for the analysis

^*^The genes that are up-regulated in both larvae of Cq_SP-R and Cq_NiH-R strains

^#^The genes that are up-regulated in both larvae and adults of Cq_SP-R and/or Cq_NiH-R strains

**Table 4:** The relative level of gene expression of *cytochrome P450* genes in both larvae and adult stages of the deltamethrin selected Cq_NiH-R and its parental Cq_NiH strains

Stage of mosquito	Transcript ID^a^	Gene	Relative gene expression ± SE^b^		Ratio^c^	*P*-value^d^
			Cq_NiH (parental strain)	Cq_NiH-R (resistant strain)		
*Cytochrome P450* genes involved in up-regulation						
Larva	CPIJ018943	*CYP4C52v1*	1.26 ± 0.04	2.49 ± 0.07	2.0	< 0.0001
	CPIJ011127	CYP4H34* ^#^	0.39 ± 0.06	1.99 ± 0.05	5.1	< 0.0001
	CPIJ005959	CYP6AA7* ^#^	0.41 ± 0.05	9.92 ± 0.06	24.2	< 0.0001
	CPIJ005955	CYP6P14* ^#^	0.57 ± 0.05	10.43 ± 0.05	18.2	< 0.0001
	CPIJ012470	CYP9AL1* ^#^	0.64 ± 0.06	1.82 ± 0.06	2.8	< 0.0001
	CPIJ010546	CYP9J34*	0.63 ± 0.09	15.11 ± 0.07	24.1	< 0.0001
	CPIJ010537	CYP9J45* ^#^	0.74 ± 0.05	3.41 ± 0.07	4.6	< 0.0001
	CPIJ014218	CYP9M10* ^#^	0.39 ± 0.05	1.49 ± 0.04	3.8	< 0.0001
Adult	CPIJ018943	*CYP4C52v1*	0.95 ± 0.08	1.51 ± 0.09	1.6	< 0.0001
	CPIJ005959	*CYP6AA7*^#^	0.28 ± 0.06	3.60 ± 0.06	13.0	< 0.0001
	CPIJ005955	*CYP6P14*^#^	0.72 ± 0.08	12.94 ± 0.08	18.0	< 0.0001
	CPIJ012470	*CYP9AL1*^#^	0.91 ± 0.08	2.42 ± 0.09	2.7	< 0.0001
	CPIJ010537	*CYP9J45*^#^	0.31 ± 0.06	2.26 ± 0.07	7.3	< 0.0001
	CPIJ014218	*CYP9M10*^#^	1.10 ± 0.06	4.11 ± 0.08	3.7	< 0.0001
*Cytochrome P450* genes involved in down-regulation						
Adult	CPIJ011127	*CYP4H34*	4.08 ± 0.07	2.68 ± 0.08	− 1.5	< 0.0001
	CPIJ010546	*CYP9J34*	4.95 ± 0.06	2.80 ± 0.06	− 1.8	< 0.0001

^a^The transcript ID number from the vectorbase of the *Cx. quinquefasciatus* genome sequence (http://cquinquefasciatus.vectorbase.org/)

^b^The relative level of gene expression represents the ratio of the gene expression in each resistant strain compared with that in the susceptible Cq_Sus strain. The relative level of gene expression for Cq_Sus is 1.0

^c^The ratio of the relative gene expression in each deltamethrin selected strain compared its parental strain

^d^The independent samples *t*-test is used to calculate the difference in relative gene expression between the deltamethrin selected strain and its parental strain using SPSS version 17.0 (SPSS Inc., Chicago, USA) for the analysis

^*^The genes that are up-regulated in both larvae of Cq_SP-R and Cq_NiH-R strains

^#^The genes that are up-regulated in both larvae and adults of Cq_SP-R and/or Cq_NiH-R strains

In the larvae stage, *CYP6AA7* and *CYP9J34* genes exhibited a significant overexpression with the highest expression ratios of > 24-fold, followed by the *CYP6P14* gene (18.2-fold) in the Cq-NiH-R strain. The *CYP9J34* gene was also overexpressed with a high expression ratio of > sevenfold in the larvae of the Cq_SP-R strain. In the adult stage, the *CYP6P14* genes exhibited overexpression (18.0-fold), followed by *CYP6AA7* (13.0-fold) and *CYP9J45* (7.3-fold) in the Cq_NiH-R strain. After exposure to deltamethrin, the larvae of *Cx. quinquefasciatus* showed an increase in the expression of *cytochrome P450* genes. However, none of these genes were found to be down-regulated in the larvae of the Cq_NiH-R strain. On the other hand, six genes (*CYP4C52v1*, *CYP6AA7*, *CYP6P14*, *CYP9AL1*, *CYP9J45*, *CYP9M10*) were down-regulated in adult stage of Cq_SP-R strain.

### Partial sequencing of the *Culex quinquefasciatus*-*vgsc* gene

To determine the point mutations in the *vgsc* gene involved in resistance to deltamethrin, four fragments of the *Cx. quinquefasciatus*-*vgsc* gene covering four domains (I–IV) were partially sequenced using direct sequencing. Those fragment sequences of each mosquito strain were submitted to the GenBank database (Genbank: PQ720348–52 for the IS2–IS6 region of domain I, PQ678660–64 for the IIS1–IIS6 region of domain II, PQ720353–57 for the IIIS1–IIIS6 region of domain III and PQ720358–62 for the IVS1–IVS6 region of domain IV). The *kdr* L1014F mutation was observed in the Cq_SP and the deltamethrin-resistant selected strain of Cq_SP-R (Fig. 1). However, the *kdr* L1014F mutation was not found in other deltamethrin-resistant Cq_NiH-R or its parental Cq_NiH strains. Interestingly, two more non-synonymous mutations, V240M and new L925F, were identified in the Cq_SP-R strain (Fig. 1). Genotyping of the V240M, L925F and *kdr* L1014F mutations was then conducted for six individuals of the Cq_SP-R strain using direct sequencing (Table 5). All individuals of the Cq_SP-R strain were heterozygous (L/F925) genotype for the L925F mutation and homozygous mutant (F/F1014) genotype for the *kdr* L1014F mutation (Table 5). For the V240M mutation, homozygous mutant (M/M240) and heterozygous (V/M240) genotypes were observed at the frequency of 33.3 and 66.7% in the Cq_SP-R strain, respectively.

**Table 5:** Genotyping of the V240M, L925F and *kdr* L1014F mutations of the *vgsc* gene for six individual samples from the deltamethrin-resistant strain of Cq_SP-R using direct sequencing

*vgsc* mutation	No. of tested	Genotype frequency, *n* (%)			Allele frequency (%)	
		Homozygous wild type	Heterozygous	Homozygous mutant	Wild type	Mutant
V240M	6	0 (0.0)	4 (66.7)	2 (33.3)	33.3	66.7
L925F	6	0 (0.0)	6 (100.0)	0 (0.0)	50.0	50.0
L1014F	6	0 (0.0)	0 (0.0)	6 (100.0)	0.0	100.0

### Genotyping of *kdr* L1014F mutation in the *Culex quinquefasciatus-vgsc* gene

Genotype frequency of the *kdr* L1014F mutation in the *vgsc* gene was conducted on both survivor and dead mosquitoes in five strains of *Cx. quinquefasciatus* after exposure to 0.05% deltamethrin WHO paper. The genotype frequency of the *kdr* L1014F mutation differed between survivor and dead mosquitoes in the Cq_SP and Cq_SP-R strains (Table 6). The homozygous mutant (F/F1014) had a higher frequency in the survivor (57.7%) than dead (15.0%) mosquitoes in the Cq_SP strain. The F1014 mutant allele frequency in the Cq_SP survivor mosquitoes (65%) was significantly higher than in the Cq_SP dead mosquitoes (20.0%) (*P* < 0.0001). In the Cq_SP-R strain, no dead mosquito was found after exposure to 0.05% deltamethrin WHO paper (Table 6). Only survivor mosquitoes were randomly genotyped for the *kdr* L1014F mutation. A high homozygous mutant (F/F1014) genotype with a frequency of 87.2% was observed in the survivor Cq_SP-R mosquitoes. Interestingly, the F1014 mutant allele showed a significantly higher frequency in the survivor Cq_SP-R (91%) than Cq_SP (65%) mosquitoes (*P* < 0.0001). The Cq_NIH and Cq_NIH-R mosquitoes were examined on survivor and dead specimens, however, only the homozygous wild type (L/L1014) was detected in both strains. Similarly, in the Cq_Sus susceptible strain, only the homozygous wild type (L/L1014) was detected in both survivor and dead mosquitoes.

**Table 6:** Genotype and allele frequency of the *kdr* L1014F mutation in each strain of *Culex quinquefasciatus* within survivor and dead mosquitoes after exposure to 0.05% deltamethrin WHO paper

Strain	Status	No. of tested	*kdr* L1014F genotyping No. of mosquitoes (% frequency)			Allele frequency (%)		*P-value*
			L/L	L/F	F/F	L	F	
Cq_SP	Survivor	26	7 (26.9)	4 (15.4)	15 (57.7)	35.0	65.0	< 0.0001*
	Dead	20	15 (75.0)	2 (10.0)	3 (15.0)	80.0	20.0	
	Total	46	22 (47.8)	6 (13.1)	18 (39.1)	54.0	46.0	
Cq_SP-R	Survivor	39	2 (5.1)	3 (7.7)	34 (87.2)	9.0	91.0	ND
	Dead	0	ND	ND	ND	ND	ND	
	Total	39	2 (5.1)	3 (7.7)	34 (87.2)	9.0	91.0	
Cq_NiH	Survivor	23	23 (100.0)	0 (0.0)	0 (0.0)	100.0	0.0	1.000
	Dead	24	24 (100.0)	0 (0.0)	0 (0.0)	100.0	0.0	
	Total	47	47 (100)	0 (0.0)	0 (0.0)	100.0	0.0	
Cq_NiH-R	Survivor	17	17 (100)	0 (0.0)	0 (0.0)	100.0	0.0	1.000
	Dead	17	17 (100)	0 (0.0)	0 (0.0)	100.0	0.0	
	Total	34	34 (100)	0 (0.0)	0 (0.0)	100.0	0.0	
Cq_Sus	Survivor	2	2 (100.0)	0 (0.0)	0 (0.0)	100.0	0.0	1.000
	Dead	34	34 (100.0)	0 (0.0)	0 (0.0)	100.0	0.0	
	Total	36	36 (100.0)	0 (0.0)	0 (0.0)	100.0	0.0	

^*^The mutant F allele showed significantly higher frequencies in survivor mosquitoes than those in dead mosquitoes (*P* < 0.0001) using Fisher’s exaction test. L and F are concatenated bases representing leucine and phenylalanine, respectively

*L/L* homozygous wild type genotype for L1014F mutation, *L/F* heterozygous genotype for L1014F mutation, *F/F* homozygous mutant genotype for L1014F mutation, *ND* not determined

### Haplotype of *CYP9M10* gene in *Culex quinquefasciatus*

The *CYP9M10* gene exhibited overexpression in deltamethrin-resistant selected mosquitoes, encompassing both larvae and adults of Cq_NiH-R and larvae of Cq_SP-R, in comparison to their parents (Cq_NiH and Cq_SP) and susceptible (Cq_Sus) mosquitoes (Tables 3–4). The haplotype of the *CYP9M10* gene was assessed in both survivor and dead mosquitoes within each strain of *Cx. quinquefasciatus* after exposure to 0.05% deltamethrin WHO paper. Only two haplotypes of the *CYP9M10* gene, the *CuRE1* (*Culex repetitive element 1*) inserted and duplicated [D-Cu(+)], and the *CuRE1* non-inserted wild type [Cu(−)], were identified across five mosquito strains (Table 7). The homozygous genotype for the *CuRE1* non-inserted wild type [Cu(−)/Cu(−)] was prevalent in each strain. The heterozygous genotype for the *CuRE1* inserted and duplicated, and the *CuRE1* non-inserted wild type [D-Cu(+)/Cu(−)] was detected with a total frequency of 13.3% and 8.3% in Cq_NiH (*n* = 30) and Cq_NiH-R (*n* = 36), respectively. Both the survivor and dead mosquitoes of the Cq_NiH strain demonstrated a similar frequency of the heterozygous genotype for the D-Cu(+)/Cu(−), but only the survivor mosquitoes of the Cq_NiH-R strain exhibited this heterozygous genotype. No heterozygous genotype for the D-Cu(+)/Cu(−) was observed in the dead group of the Cq_NiH-R strain. However, the Cq_SP, Cq_SP-R and Cq_Sus strains were exclusively homozygous for the Cu(−)/Cu(−) in both the survivor and dead groups.

**Table 7:** Genotype frequency of the *CYP9M10* gene in the *Culex quinquefasciatus* within survivor and dead adult mosquitoes after exposure to 0.05% deltamethrin WHO paper

Strain	Status	No. of tested	*CYP9M10* genotyping No. of mosquitoes (% frequency)	
			Cu(−)/Cu(−)	D-Cu(+)/Cu(−)
Cq_SP	Survivor	17	17 (100.0)	0 (0.0)
	Dead	18	18 (100.0)	0 (0.0)
	Total	35	35 (100.0)	0 (0.0)
Cq_SP-R	Survivor	24	24 (100.0)	0 (0.0)
	Dead	0	ND	ND
	Total	24	24 (100.0)	0 (0.0)
Cq_NIH	Survivor	24	21 (87.5)	3 (12.5)
	Dead	6	5 (83.4)	1 (16.6)
	Total	30	26 (86.7)	4 (13.3)
Cq_NIH-R	Survivor	19	16 (84.2)	3 (15.8)
	Dead	17	17 (100.0)	0 (0.0)
	Total	36	33 (91.7)	3 (8.3)
Cq_Sus	Survivor	2	2 (100.0)	0 (0.0)
	Dead	22	22 (100.0)	0 (0.0)
	Total	24	24 (100.0)	0 (0.0)

Cu(−), *CuRE1* non-inserted (wild type); D-Cu(+), CuRE1-inserted and duplicated (strong type).

*ND* not determined

## Discussion

This study has demonstrated, for the first time, the overexpression of *CYP450* genes conferring pyrethroid resistance in the populations of *Cx. quinquefasciatus* collected from Thailand specifically within the Chiang Mai and Bangkok Provinces. These locations served as the original sites for collecting the Cq_SP and Cq_NiH mosquito strains, respectively. This study explored the mechanisms that contribute to insecticide resistance in populations of *Cx. quinquefasciatus* in Thailand, which is critically important for public health in the region. With the rise in resistance to pyrethroid, especially deltamethrin, becoming more pronounced [4, 10, 19], it is imperative to comprehend the genetic and biochemical factors contributing to this resistance increase. Understanding these factors is essential for developing effective strategies for vector control [5–8].

This study underscored the two primary mechanisms of resistance: metabolic resistance mediated by cytochrome P450 and target site insensitivity associated with *kdr* mutations. These mechanisms have significant potential to impact the effectiveness of existing insecticide-based interventions [5–7]. A comprehensive understanding of these mechanisms will enable public health officials to modify and enhance vector control programs effectively. This approach ensures the sustained effectiveness of strategies aimed at reducing mosquito populations. This could play a critical role in reducing the transmission of mosquito-borne diseases, including lymphatic filariasis and other diseases associated with *Culex* species [3, 5–7]. This study ultimately supports the sustainability of public health initiatives in Thailand, contributing to preserving community health and enhancing disease management strategies [2, 6, 7]. These efforts are essential in effectively addressing the challenges posed by evolving resistance.

Several *CYP450* genes, *CYP4C52v1*, *CYP4H34*, *CYP6AA7*, *CYP6P14*, *CYP9AL1*, *CYP9J34*, *CYP9J45,* and *CYP9M10*, were overexpressed in Cq_NiH-R and Cq_SP-R in larval and adult stages compared to their parent and susceptible strains. The Cq_NiH-R mosquitoes, a deltamethrin-selected strain originally collected from Bangkok Province, exhibited no *kdr* L1014F mutation. All eight *CYP450* genes were significantly overexpressed in the larval stage of the Cq_NiH mosquitoes, while five genes, *CYP6AA7*, *CYP6P14, CYP9AL1, CYP9J45,* and *CYP9M10*, showed overexpression in both larval and adult stages. This suggested that the overexpression of *CYP450* genes might be the major mechanism involving deltamethrin resistance throughout the Cq_NiH-R mosquito’s lifespan. However, only two genes, *CYP4H34* and *CYP9J34,* showed significant overexpression in both larval and adult stages of the Cq_SP-R strain. The overexpression of *CYP6AA7* and *CYP9J34* genes was previously found in both larva and adult permethrin-resistant (PerRes) strains [33], while the *CYP6AA7* were significantly overexpressed in only adult of permethrin-resistant HAmCq^G8^ mosquitoes [24]. The *CYP450* genes may be involved in insecticide pressure in different developmental stages and populations of mosquitoes, with some being specific to particular developmental stages and others protecting the insect’s life cycle [34].

The overexpression of functional *CYP450* genes in resistant insects is a common mechanism of insecticide resistance, and multiple over-transcribed *CYP450* genes belonging to the *CYP6* and *CYP9* families have been identified in pyrethroid resistant *Cx. quinquefasciatus* mosquito strains or populations [13, 14, 18, 33, 35, 36]. Among these eight overexpressed *CYP450* genes, *CYP4C52v1*, *CYP6AA7*, *CYP6P14*, *CYP9J34*, *CYP9J45* and *CYP9M10,* it has been demonstrated that decreased expression of these *CYP450* genes corresponded with the reduced level of insecticide resistance to permethrin via RNA interference (RNAi) analysis [14, 37]. Five of those *CYP450* genes, *CYP6AA7*, *CYP6P14*, *CYP9J34*, *CYP9J45* and *CYP9M10,* have been functionally confirmed by playing an important role in permethrin degradation pathways using heterogeneous expression and in vitro metabolism study [15, 38].

The *CYP9M10* gene has also demonstrated a role in conferring permethrin resistance in *Cx. quinquefasciatus* mosquitoes using the TALENs and CRISPR techniques [39]. The *CYP9M10* gene has been well characterized concerning permethrin resistance only in the larval stage [39–41]. However, the *CYP9M10* gene was overexpressed with a similar expression ratio (~ fourfold) in the larvae and adults of the Cq_NiH-R strain indicating this gene conferred deltamethrin resistance in both mosquito stages. Although the duplicated *CYP9M10* haplotype associated with strong larval resistance [39] was also detected in adults of Cq_NiH-R and its parent, this haplotype likely has little effect on the mortality rate in the adult stage, considering the low allele frequency in both strains.

The higher level of resistance to deltamethrin in the Cq_SP-R than in the Cq_NiH-R might be due to the Cq_SP-R strain carried by the common *kdr* L1014F. In the Cq_SP-R strain, the *kdr* L1014F mutation is associated with resistance against deltamethrin, typer II pyrethroid, and is likely confers cross-resistance to permethrin, type I pyrethroid. Insects carrying *kdr* mutations often exhibit cross-resistance to both type I and type II pyrethroids [16]. The L1014F mutation is found worldwide in *Cx. quinquefasciatus* mosquitoes and other insect species have been functionally confirmed to be responsible for *kdr* in an in vitro expression system, *Xenopus* oocytes [16]. In the absence of selection pressure, the frequency of the *kdr* L1014F allele in houseflies, which was highly resistant, was relatively constant over time. In contrast, there was a fitness cost for the *super-kdr* (M918T + L1014F) allele, which significantly decreased over time [42]. Moreover, we detected two non-synonymous mutations (V240M and L925F) in Cq_SP-R mosquitoes, suggesting they may play a role in deltamethrin resistance.

A small proportion of homozygous wide type L/L1014 (5.1%) and heterozygous L/F1014 (7.7%) mosquitoes of Cq_SP-R were alive after exposure to 0.05% deltamethin WHO paper [22] due to the additional V240M and L925F mutations in *vgsc* gene and/or metabolic resistance mechanism. The V240M was previously reported in the pyrethroid-resistant *Cx. quinquefasciatus* from Myanmar and all mosquitoes were heterozygous (V/M240) genotype analyzed using next-generation sequencing [17]. The L925F has not been reported in any insect [17, 19]. The role of the V240M and novel L925F mutations in resistance requires further investigation. The mutations L932F and I936V in the Brazilian strains of *Cx. quinquefasciatus*, respectively, have been previously identified to be associated with pyrethroid resistance [17]. Two other non-synonymous mutations, V978E and D992E, have been identified in deltamethrin-resistant individuals from Chiang Mai Province, Thailand [19]. The Cq_SP-R strain also exhibited the overexpression of multiple *CYP450* genes but at a lower level than the Cq_NiH-R strain. This result indicated that the target site VGSC alteration, particularly *kdr* L1014F mutation, is not the only mechanism involved in deltamethrin resistance in the Cq_SP-R strain. The overexpression of multiple *CYP450* genes may play a role in metabolic resistance in this deltamethrin-selected strain.

The interaction between two pyrethroid resistance loci, *kdr* and cytochrome P450 monooxygenases, has previously been documented in the pyrethroid-resistant C*x. quinquefasciatus* strains of Cq_CM-R and JPAL originating from Thailand and Saudi Arabia, respectively [4, 43, 44]. While previous studies utilized the inhibitor, piperonyl butoxide (PBO), to demonstrate the involvement of CYPs in pyrethroid-resistant mosquitoes, PBO does not allow for accurately quantifying the contribution of CYPs to the observed resistance. This study provides additional evidence supporting the contribution of the overexpression of multiple *CYP450* genes in insecticide resistance. It provides new light on the interactions between the *CYP4H34* and *CYP9J34* genes and the *kdr* L1014F mutation in conferring resistance to deltamethrin.

In addition, it would be valuable to investigate the other *CYP450* genes associated with metabolic resistance mediated by cytochrome P450s in the deltamethrin-resistant strain of *Cx. quinquefasciatus*. A comprehensive analysis of *kdr* mutations in these mosquitoes, covering the entire length of the *Cx. quinquefasciatus-vgsc* gene, could provide important insights.

## Conclusions

This study highlights the overexpression of multiple *CYP450* genes, with or without the *kdr* L1014F mutation, resulting in a high level of resistance to deltamethrin in Thai populations of *Cx. quinquefasciatus*. The overexpression of multiple *CYP450* genes alone contributes less to resistance. The effect of the combined mechanisms, *CYP450-* and *kdr-*mediated resistance, was significantly higher resistance for deltamethrin in Thai populations of *Cx. quinquefasciatus*. According to our findings, it is recommended to avoid the use of deltamethrin in regions where *Cx. quinquefasciatus* populations demonstrate a high frequency of *kdr* L1014F mutation, alongside the observed overexpression of *CYP450* genes.

## Data Availability

Please contact the corresponding author for data requests.
